# Molecular Identification and In Silico Protein Analysis of a Novel *BCOR-CLGN* Gene Fusion in Intrathoracic *BCOR*-Rearranged Sarcoma

**DOI:** 10.3390/cancers15030898

**Published:** 2023-01-31

**Authors:** Yi-Che Chang Chien, Kristóf Madarász, Szilvia Lilla Csoma, János András Mótyán, Hsuan-Ying Huang, Gábor Méhes, Attila Mokánszki

**Affiliations:** 1 Department of Pathology, Faculty of Medicine, University of Debrecen, H-4032 Debrecen, Hungary; 2 Department of Biochemistry and Molecular Biology, Faculty of Medicine, University of Debrecen, H-4032 Debrecen, Hungary; 3 Department of Pathology, Kaohsiung Chang Gung Memorial Hospital, Chang Gung University College of Medicine, Kaohsiung 83301, Taiwan

**Keywords:** *BCOR*-rearranged sarcoma, immunohistochemistry, *BCOR-CLGN* gene fusion, next-generation sequencing, in silico protein analysis, calmegin, BCL6 corepressor

## Abstract

**Simple Summary:**

*BCOR* (*BCL6* corepressor)-rearranged sarcoma (BRS) is a rare sarcoma entity with a predominantly *BCOR-CCNB3* fusion. In this paper, we present an index case of BRS with a novel *BCOR-CLGN* (calmegin) gene fusion that was first identified by next-generation sequencing and then verified by Sanger sequencing. We also carried out in silico protein analysis to demonstrate the 3D structure of the chimera protein. We concluded that, due to its heterogeneity, molecular ancillary tests serve as powerful tools to discover such unusual variants. The fusion protein used in the in silico analysis is an appropriate approach to understanding the exact pathogenesis of such a rare variant.

**Abstract:**

*BCOR* (*BCL6* corepressor)-rearranged sarcomas (BRSs) are a heterogeneous group of sarcomas previously classified as part of the group of “atypical Ewing” or “Ewing-like” sarcomas, without the prototypical *ESWR1* gene translocation. Due to their similar morphology and histopathological features, diagnosis is challenging. The most common genetic aberrations are *BCOR-CCNB3* fusion and *BCOR* internal tandem duplication (ITD). Recently, various new fusion partners of *BCOR* have been documented, such as *MAML3*, *ZC3H7B*, *RGAG1*, and *KMT2D*, further increasing the complexity of such tumor entities, although the molecular pathogenetic mechanism remains to be elucidated. Here, we present an index case of intrathoracic BRS that carried a novel *BCOR-CLGN* (calmegin) gene fusion, exhibited by a 52-year-old female diagnosed initially by immunohistochemistry due to the positivity of a BCOR stain; the fusion was identified by next-generation sequencing and was confirmed by Sanger sequencing. In silico protein analysis was performed to demonstrate the 3D structure of the chimera protein. The physicochemical properties of the fusion protein sequence were calculated using the ProtParam web-server tool. Our finding further broadens the fusion partner gene spectrum of BRS. Due to the heterogeneity, molecular ancillary tests serve as powerful tools to discover these unusual variants, and an in silico analysis of the fusion protein offers an appropriate approach toward understanding the exact pathogenesis of such a rare variant.

## 1. Introduction

Aberrant *BCOR* (BCL6 corepressor) expression has been found in a variety of tumor types, including clear cell sarcoma of the kidney, endometrial sarcoma, rhabdomyosarcoma, and central nervous system and myeloid tumors [1,2,3]. Among these, *BCOR-*rearranged small blue round cell sarcoma (BRS), which commonly occurs in the bones of young patients, represents a rare soft tissue tumor entity and shares morphological similarities with Ewing sarcoma (ES) without the *EWSR1* rearrangement described first by Pierron et al. [4]. The *BCOR* gene is located in the Xp11.4 chromosome region; it facilitates BCL6-regulated transcriptional repression via epigenetic signal transduction mechanisms [5,6] to mediate the apoptotic and oncogenic activities of the cells [7]. The epigenetic modification of histones requires *BCOR* bounding to the polycomb-group RING finger homolog (PCGF) of polycomb-repressive complex 1 (PRC1) [8], via the ubiquitin-like fold discriminator (PUFD) domain [9]. PRC1 functions by adding a ubiquitin moiety to the histone H2A at Lys119 (H2AK119). PRCs silence several genes, including the *HOX* group genes [8,10]. *BCOR* participates in one of the non-canonical PRC1 complexes, PRC1.1 [11].

In a previous study, Wang et al. found that the presence of BCOR at BCOR-responsive targets is crucial for the recruitment of the PRC1.1 complex, the maintenance of H3K27me3, and the recruitment of cPRC1 complexes. Without the C-terminal domain, the protein does not interact with KDM2B, PCGF1, and RNF2; the C-terminus of BCOR is both necessary and sufficient for BCOR targeting. The linker and PUFD regions are critical for recruiting BCOR and maintaining polycomb domains at the targets [12].

The most common *BCOR* aberrations in BRS are internal tandem duplication (ITD) and translocation, causing gene fusion; for the latter, the most common fusion partner is *CCNB3* in the Xp11.22 chromosome region due to paracentric inversion, which links the *BCOR* coding sequence to exon 5 of the *CCNB3* gene. The additional stretch of amino acids (ITD) in the PUFD domain might interfere with PCGF1 binding and, thus, could affect PRC1-related epigenetic modifications [13,14,15].

BRS is currently classified under the category of “undifferentiated small round cell sarcoma of soft tissue and bone”, based on the latest WHO classification (2020) and represents approximately 15% of cases within this category. Despite the morphological resemblance to Ewing sarcoma (ES), the genomic and transcriptomic profiles of BRSs showed that they are genetically distinct entities [16]. Nevertheless, the therapeutic strategy for BRS is similar to ES, with complete surgical resection being the most important treatment. Meanwhile, BRSs also show a significant response to the ES chemotherapeutic protocol. In the area of precision oncology, due to advances in molecular diagnostic methodology, particularly next-generation sequencing (NGS), BRSs with variable fusion partner genes have been discovered, including *MAML3*, *ZC3H7B*, *RGAG1*, and *KMT2D* [17,18,19].

The aims of our study were (i) to present the histological and immunohistochemical (IHC) staining features in a case of BRS with unusual intrathoracic manifestation, (ii) to design a custom RNA-based NGS panel, including genes that may be involved in the pathogenesis of these rare entities of sarcomas, (iii) to identify somatic gene mutations (SNVs and indels) and gene fusions using NGS, (iv) to confirm the detected translocation using fluorescence in situ hybridization (FISH), (v) to verify gene fusions after reverse transcription (RT) using classic bidirectional Sanger sequencing, and (vi) to perform an in silico protein analysis to predict its function in the detected fusion protein. For this purpose, histologic examination, IHC, *BCOR* FISH, RNA isolation, RT, Sanger sequencing, an NGS gene panel analysis targeting 54 genes (Archer FusionPlex custom panel, Illumina MiSeq platform), and in silico protein analyses were performed. Here, we present a novel case of intrathoracic soft tissue somatic carried *BCOR-CLGN* (calmegin) gene fusion, which further expands the genetic diversity of BRS. The *CLGN* gene encodes the calmegin protein, but its oncological significance is currently unknown. 

## 2. Materials and Methods

### 2.1. Case Selection Criteria

The retrospective patient population is derived from the Department of Pathology at the University of Debrecen, from the period of January 2015–December 2022. Cases diagnosed as “atypical Ewing sarcoma” without *EWSR1* rearrangement being detected by FISH were investigated. Due to its rarity, we found only one case that met these criteria. 

For research purposes, this patient consented and agreed, according to the ethical guidelines of the University of Debrecen, to the use of tissue blocks and clinical information for this report. All protocols have been approved by the authors’ respective Institutional Review Boards for human subjects (IRB reference number: 60355-2/2016/EKU and IV/8465-3/2021/EKU). This study was managed according to the Declaration of Helsinki.

### 2.2. Histology and Immunohistochemistry

Hematoxylin and eosin (H&E)-stained slides were analyzed by pathology specialists. Histological evaluation and interpretation were conducted according to WHO classification.

After the sectioning of 4 μm slides from formalin-fixed, paraffin-embedded (FFPE) blocks, deparaffinization in xylene, and rehydration in a series of decreasing concentrations of ethanol were performed. Antigen retrieval using either the Bond Epitope Retrieval Solution 1 (pH~6) or the Bond Epitope Retrieval Solution 2 (pH~9) (Leica Biosystems, Wetzlar, Germany) was carried out at 99–100 °C for 20–30 min. The slides were then treated with cytokeratin (1:450, clone OSCAR, Cell Marque, Rocklin, CA, USA), WT-1 (1:700, clone WT49, Leica Biosystems, Wetzlar, Germany), NUT (1:200, clone C52B1, Cell Signaling Technology, Danvers, MA, USA), CD56 (1:400, clone 123C3, DAKO, Agilent Technologies, Santa Clara, CA, USA), BCOR (1:400, clone bsb-128, Bio SB Incorporation, Goleta, CA, USA), CD99 (1:200, clone 12E7, DAKO, Agilent Technologies, Santa Clara, CA, USA), INSM1 (1:600, clone A-8, Santa Cruz Biotechnology, Dallas, TX, USA), NKX2.2 (1:400, clone EP336, Epitomics, Abcam, Cambridge, UK), SATB2 (1:200, clone EP281, Epitomics, Abcam, Cambridge, UK), TLE1 (1:450, 1F5, Cell Marque, Rocklin, CA, USA), SS18-SSX (1:400, clone E9X9V, Cell Signaling Technology, Danvers, MA, USA), Brg1 (1:150, clone GT2712, Thermo Fisher Scientific, Waltham, MA, USA), desmin (1:200, clone D33, DAKO, Agilent Technologies, Santa Clara, CA, USA), and MIB-1 (1:100, clone MIB-1, Dako, Agilent Technologies, Santa Clara, CA, USA) separately. Immunostaining was conducted with the Leica BOND-MAX™ autostainer and the peroxidase/DAB Bond™ Polymer Refine detection system (Leica Biosystems, Wetzlar, Germany) was utilized for visualization purposes.

### 2.3. Fluorescence and In Situ Hybridization

FISH was performed on 5 µm-thick sections of the FFPE block, with *EWSR1* (MetaSystems, Altlussheim, Germany) and *BCOR* (bacterial artificial clones flanking the *BCOR* (RP11-91I16 and RP11-665O2) on the X chromosome, from the BACPAC Resources Center, Emeryville, CA, USA) break-apart probes separately, using a modified manufacturer’s protocol. Deparaffinized sections (Q Path Safesolv, VWR, Debrecen, Hungary) were treated with protease solution (MetaSystems, Altlussheim, Germany). Slide and probe co-denaturation was carried out at 75 °C for 10 min, while hybridization was at 37 °C in a moist chamber for 18 h (StatSpin ThermoBrite, Abbott Molecular, Des Plaines, IL, USA). Post-hybridization washes were performed with 2× saline–sodium citrates (SSC) for 5 min. The slides were then washed with 0.4× SSC at 74 °C for 3 min and 2× SSC/0.05% Tween 20 for 2 min. After washing, the nuclei were counterstained with 4′-6′ diamidino-2-phenylindole (DAPI, MetaSystems, Altlussheim, Germany). Scoring was performed using a Zeiss Axio Imager Z2 (Carl Zeiss, Cambridge, UK) fluorescence microscope. The images were captured and analyzed using ISIS software v.5.5.4. (MetaSystems, Altlussheim, Germany). 

### 2.4. RNA Isolation

Genomic tumor-derived RNA (tdRNA) was extracted from FFPE tissues using the ReliaPrep FFPE Total RNA Miniprep System (Promega, Madison, WI, USA) according to the manufacturer’s instructions. The concentration of tdRNA was measured with the Qubit™ RNA HS Assay Kit, using a Qubit 4.0 fluorimeter (Thermo Fisher Scientific, Waltham, MA, USA).

### 2.5. Next-Generation Sequencing

For NGS library preparation, an RNA-based Archer FusionPlex custom gene panel (Archer DX, Boulder, CO, USA) specific for sarcomas, including the *EWSR1* and *BCOR* genes, was applied to identify the SNVs, indels, and gene fusions. This solution uses anchored primers with known translocation partners and reverse primers that hybridize into sequencing adapters to detect breakpoints and partners. A total of 100–250 ng of RNA was loaded into the assay. cDNA was taken after the first-strand synthesis and run in a quantitative RT-PCR pre-sequencing QC assay. The pre-sequencing quantitative reverse-transcription PCR assay was performed in duplicate on cDNA, using primers targeting the control gene transcripts of VCP and using 35 cycles to determine the amount of intact RNA in a given sample. The final libraries were quantified with a KAPA library quantification kit (Roche, Basel, Switzerland), diluted to a final concentration of 4 nM, and pooled by equal molarity.

For sequencing on the MiSeq System (MiSeq Reagent kit, version 3, 600 cycles), the libraries were denatured using 0.2 nM NaOH and diluted to 40 pM with hybridization buffer (Illumina, San Diego, CA, USA). The final loading concentration was 8 pM libraries and 5% PhiX. Captured libraries were sequenced in a multiplexed fashion with a paired-end run, to obtain 2 × 150 bp reads, with a depth of coverage of at least 500×. Sequencing was conducted according to the MiSeq instruction manual. Trimmed fastq files were generated using the MiSeq reporter (Illumina, San Diego, CA, USA) and were uploaded to the Archer Analysis v7 website (Archer DX, Boulder, CO, USA). For alignment, the human reference genome GRCh37 (equivalent UCSC version hg19) was built. Translocations were stated at a fusion sequence of over 5 reads, with reads from gene-specific primers comprising at least 10% of the total reads. Gene fusion frequency was calculated for both the fusion transcript reads and the total reads ratio.

### 2.6. Sanger Sequencing

For confirming the *BCOR* fusion, detected by NGS, and knowing the breakpoints, Sanger sequencing was performed. To exclude the possibility of an analytical mistake, another RNA isolation method was also used to fulfill the Sanger sequencing (Trizol reagent, Thermo Fisher Scientific, Waltham, MA, USA). RNA quality was determined via eukaryote total RNA nano assays.

For Sanger sequencing, a reverse transcription-polymerase chain reaction (RT-PCR) was carried out. The cDNA quality was tested for the phosphoglycerate kinase 1 (PGK1) housekeeping gene (247 bp of amplified product). One microgram of total RNA was used for cDNA synthesis with a SuperScript ^®^ III First-Strand Synthesis Kit (Invitrogen, Carlsbad, CA). RT-PCR was performed using the Advantage-2 PCR kit (Clontech, Mountain View, CA, USA) for 32 cycles at an annealing temperature of 64 °C. Two pairs of primers were used for excluding analytical problems, which were as follows: *BCOR* exon 15 forward primers: 5′-GGTGGAATTCACGAACGAAA-3′ (F1), 5′-GAATTCACGAACGAAATTCAGA-3′ (F2), and *CLGN* exon 9 reverse primers: 5′-GGATAAATTTTGGTTCATCATCAAG-3′ (R1) and 5′-ATCATCAAGCCAGCCAGCA-3′ (R2). The lengths of the first and second PCR products were 170 and 150 bp, respectively. The amplified products were purified and sequenced using the Sanger method.

### 2.7. In Silico Protein Analysis

Protein information for the BCOR (Q6W2J9, BCOR_HUMAN) and CLGN (O14967, CLGN_HUMAN) proteins were obtained from the UniProt database and the RCSB Protein Data Bank. The translated BLAST:blastx web program was used to blast our nucleotide sequence [20,21,22,23,24,25,26,27,28,29]. Post-translational modifications (PTMs) of calmegin were investigated using the PhosphoSitePlus database [30]. The membrane contact probability (MCP) predictor was used to investigate the transmembrane section of the fusion protein from the calmegin protein. This section corresponds to 472–492 in calmegin and 1931–1952 in the BCOR-calmegin chimera protein [31]. The physicochemical properties were calculated using ExPASy’s ProtParam tool [32], including the theoretical isoelectric point (pI), molecular weight, the total number of positive and negatively charged residues, instability index (II) [33], aliphatic index (AI) [34], and grand average hydrophobicity (GRAVY) [35].

The instability index gives an estimate of a protein’s stability in vitro. The aliphatic index of a protein is regarded as a positive factor for the growth of thermostability of globular proteins and is specifically defined as the relative volume occupied by aliphatic side chains (alanine, valine, isoleucine, and leucine). The GRAVY score is calculated as the sum of the hydropathy values of all the amino acids, divided by the number of residues in the sequence. 

The 3D structure of the chimera protein was built using the Robetta RoseTTAFold (University of Washington, Seattle, WA, USA) [36] protein structure prediction software. Due to the limitations of RoseTTAFold, we were only able to predict a 1400 amino-acid (AA)-long region of the 2070 AA-long chimera protein. Therefore, the predicted protein encompasses the 671–2070 residues. Disorder prediction was performed by the IUPred3 web server [37]. GORIV was used for secondary structure prediction [38]. To calculate the accessible surface area (ASA), we used the Accessible Surface Area and Accessibility Calculation for Protein web server (ver. 1.2) [39]. The coordinate files of wild-type BCOR and calmegin proteins that were predicted by AlphaFold (AF) were downloaded from the AF database [40,41], while the crystal structure of BCOR’s PUFD domain was drawn from the RCSB Protein Data Bank (PDB ID: 4HPL). The crystal structure of the PUFD domain was compared to the Robetta-predicted chimera protein (PUFD-chimera-Robetta) and the PUFD domain of the BCOR protein obtained from the AlphaFold database (PUFD-BCOR-AlphaFold) [9]. Alignment of the 3D structures was performed using the PyMOL molecular graphics system (version 1.2r3pre, Schrödinger, LLC).

### 2.8. Statistical Analysis

A paired-sample *t*-test was performed to compare the IUPred3 scores between the chimera and wild-type BCOR PUFD domain. A one-way repeated measures (RM) ANOVA was performed to compare the relative ASA (0–1) and ASA (Å2) between chimera, AF predicted and experimentally determined the wild-type BCOR PUFD domains. Tukey’s multiple comparison tests were applied to determine the differences between the experimentally determined (PDB ID: 4HPL) and modeled (AlphaFold) structures of the wild-type PUFD domain and the PUFD domain of the chimera protein (Robetta).

## 3. Results

### 3.1. Case Report

**Figure 1:**
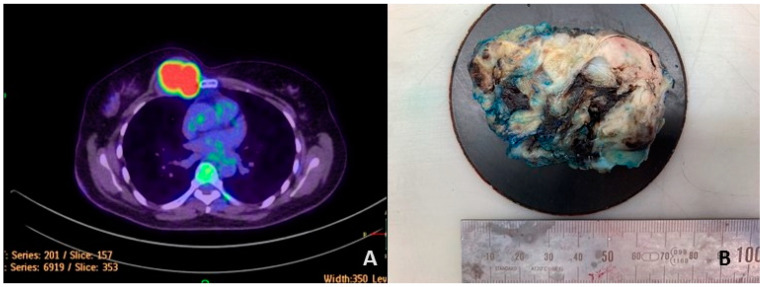
A positron emission tomography-computed tomography (PET-CT) image of the patient showed a right parasternal hypermetabolic rate tumor with thoracic wall and coastal invasion (**A**). The tumor shows a fleshy and greyish-white cut surface with a hemorrhage focus (**B**).

The index case was a 52-year-old female with a previous medical history of hysterectomy, conducted in 2021 due to an endometrial tumor (the histological diagnosis was of moderately differentiated endometrioid adenosarcoma). One year after the operation, at the postoperative follow-up, a right-side chest wall tumor was found, and positron emission tomography-computed tomography (PET-CT) revealed a right parasternal 5 × 4.5 × 3.5 cm-sized high metabolic-rate tumor with costal cartilage, pectoral muscle, and pleural invasion (Figure 1A). The result of the core needle biopsy performed in the local hospital was inconclusive. Under the clinical diagnosis of a metastatic tumor or malignant mesothelioma, tumor resection was carried out. The specimen that was received was a polypoid tumor measuring 7.5 cm at its largest diameter. The tumor showed a fleshy and greyish-white cut surface, with hemorrhage and cystic change (Figure 1B). The patient received four cycles of VIDE (vincristine, ifosfamide, doxorubicin, and etoposide) chemotherapy without radiotherapy. Up until three months of follow-up, no tumor recurrence was identified.

### 3.2. Pathological Findings

**Figure 2:**
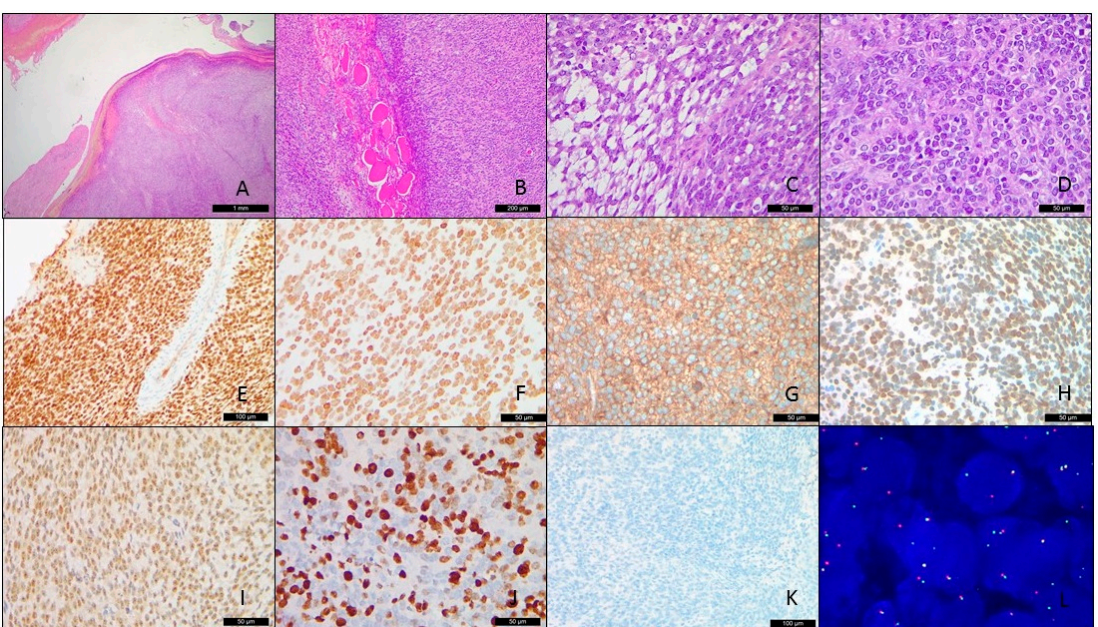
Microscopically lobulated tumor cells ((**A**), H&E, 2.5×) with infiltration into the skeletal muscle ((**B**), H&E, 10×). The ovoid- and spindle-shaped tumor cells possessed vesicular chromatin, prominent nucleoli, and minimal eosinophilic cytoplasm within the fibromyxoid stroma (**C**,**D**), H&E, 40×). The immunohistochemistry stain showed positivity for BCOR ((**E**), ×20), TLE1 (**F**), ×40), CD99 ((**G**), ×40), SATB2 ((**H**), ×40), INSM1 ((**I**), ×40) with a high MIB1 labeling index (**J**), ×40) and the pan-cytokeratin stain was negative ((**K**), 40×). The fluorescence in situ hybridization revealed *BCOR* break-apart signals (**L**).

Microscopically speaking, the H&E-stained slides showed hyper-/hypocellular small blue round cell proliferation in a lobulated pattern within the fibromyxoid-vascular stroma (Figure 2A). Infiltration of the adjacent bone and skeletal muscle was also found (Figure 2B). The tumor cells possessed ovoid to spindle-shaped nuclei with uniform, slightly vesicular chromatin features and a scant amount of clear to mildly eosinophilic cytoplasm (Figure 2C). The focal areas also revealed prominent nucleoli (Figure 2D). Tumor necrosis and hemorrhage were also noticed. Tumor mitotic activity was visible in the 18/10 high-power field. During the first round of immunohistochemical stains, the tumor cells showed positivity for CD56, CD99 (diffuse to patchy), INSM1, and NKX2.2, while they were negative for desmin, cytokeratin, SS18-SSX, and WT-1. MIB1 was up to 50%. The second IHC panel revealed diffuse, strong intranuclear positivity for BCOR, TLE1, and SATB2, and was negative for NUT. The intranuclear Brg1 staining was retained (Figure 2E–K).

### 3.3. Fluorescence In Situ Hybridization

Under the original impression of the possibility of Ewing sarcoma, an *EWSR1* FISH examination was carried out and no rearrangement of the *EWSR1* gene was identified, excluding the possibility of Ewing sarcoma. Based on the BCOR positivity via an immunohistochemical stain, the *BCOR* break-apart FISH assay was performed with a positive result, indicating *BCOR* gene rearrangement (Figure 2L).

### 3.4. NGS and Sanger Sequencing

**Figure 3:**
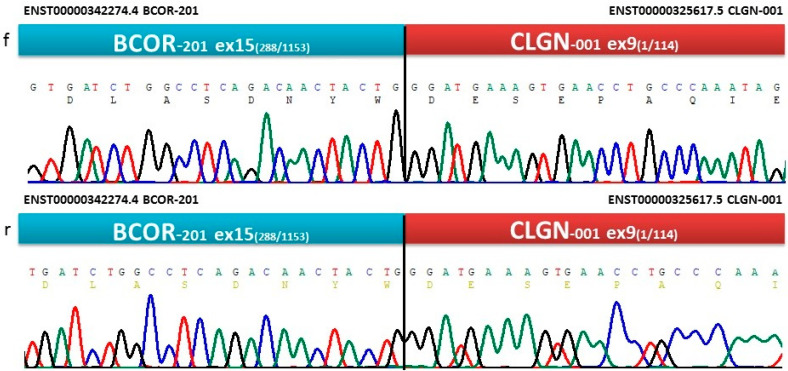
Bidirectional Sanger-sequencing electropherogram showing the nucleotide sequence at the breakpoint of *BCOR*-*CLGN* gene fusion. Note: f: forward direction, r: reverse direction.

To analyze not only the SNVs and indels but also gene fusions, NGS panels that are specific for 54 genes identified in sarcomas were applied. *BCOR*-*CLGN* gene fusion was detected (reads%: 90.8, depth: 7813, breakpoints: chrX: 39.911.366 and chr4: 141.317.359). The *BCOR* breakpoint was located in exon 15, while the *CLGN* breakpoint was affected in exon 9. Nucleotide alterations were not proven. To confirm the *BCOR*-*CLGN* fusion and identify the breakpoints, RT was followed by Sanger sequencing. The bidirectional electropherogram of the fusion genes is presented in Figure 3.

### 3.5. In Silico Protein Analysis

Translated BLAST:blastx was performed on the cDNA sequence of the *BCOR*-*CLGN* fusion gene; two proteins were found in BLASTX with a 100% match: NP_001116857.1:1–1755 BCOR (max and total score, 3606, query cover, 84%, E value, 0) and NP_001124147.1:295–610 calmegin precursor (max and total score 664, query cover 15%, E value 0). 

The full-length wild-type BCOR (UniProt ID: Q6W2J9) and calmegin (UniProt ID: O14967) proteins consist of 1755 and 610 residues, respectively. The BCOR-calmegin chimera protein consists of a total of 2070 residues, encompassing 1–1755 residues of BCOR and 296–610 residues of calmegin (Table 1). Upon fusion, the calmegin protein loses the 1–19 AA signal sequence that is responsible for its endoplasmic reticulum localization. Based on the PhosphoSitePlus database, the region of calmegin that is missing from the chimera protein (1–295) contains multiple post-translation sites, ubiquitylation on Lys101, a mono-methylation on Arg109, and acetylation sites (Lys190 and Lys291). At the same time, the transmembrane segment of calmegin (472–492) is present in the fusion protein (1931–1952 residues, according to the chimera numbering). The MCP prediction revealed that this segment of fusion protein has an average probability of 0.76 (on a scale of 0–1.0). This implies the presence of the transmembrane region close to the C-terminus of the chimera, but it remains to be determined whether the chimera protein is membrane-associated. Accordingly, 1–1755 residues of the chimera correspond to the full-length BCOR protein, while the 1756–2070 residues correspond to the truncated calmegin. At the breakpoint, two nucleotides of the *BCOR* gene (TG) and one nucleotide of the *CLGN* gene (G) encode Trp with the TGG codon; this Trp in the 1755th position corresponds to the wild-type C-terminal residue of the BCOR protein. At the breakpoint, the 1766th residue of the chimera protein is the 296th Asp of calmegin.

The physicochemical properties of the fusion protein sequence were calculated using the ProtParam web server tool. It was found that the fusion protein is composed of 2070 residues, the calculated molecular weight is 228 kDa, and the pI is 5.05. The total number of negatively charged residues (D + E) was 295, and the total number of positively charged residues (R + K) was 203 (Table 1).

The molecular weight of the chimeric protein was 15.8% and 69.3% higher than those of the wild-type BCOR and calmegin proteins, respectively. The total hydrophobicity of the chimera protein was similar, with a 3.97% and 5.38% change compared to the wild-type proteins. Nevertheless, the aliphatic index and instability index values of the chimera and the wild-type BCOR proteins are similar (there were <0.5 differences in the predicted values), while the difference is notably higher in the case of calmegin; the aliphatic index was 7.10% lower, while the instability index was 18.97% higher for the chimera. Overall, the predicted stabilities of the wild-type BCOR and the chimera proteins were highly comparable (Table 1).

The region of interest for the analyses was 1634–1748 because this is where the BCOR’s PUFD domain is located. We performed secondary structure prediction using the GORIV web server, but it did not show any considerable changes; the predicted secondary structure of the PUFD domain was identical in the wild-type BCOR and the chimera. The 296–610 region of calmegin was also predicted to have the same arrangement of secondary structural elements, even in the chimera protein.

**Figure 4:**
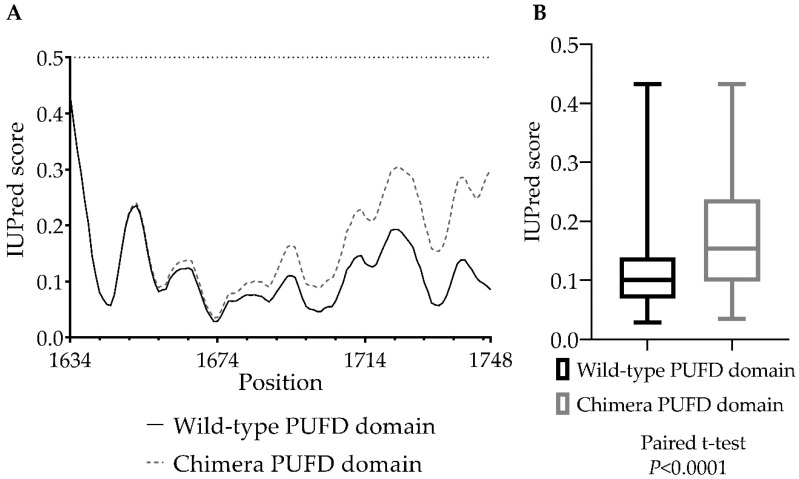
The PUFD domain’s IUPred3 long disorder analysis of wild-type BCOR and the chimera protein. (**A**): IUPred3 scores of the PUFD domain on the graph, where a score of 0 means a completely ordered protein structure and a score of 0.5 and above means a disordered protein structure. (**B**): Statistical comparison of the PUFD domain IUPred3 scores between BCOR and the chimera protein (between 1634 and 1748 residues). A significant increase was measured in the IUPred3 scores of the chimera PUFD domain, which means that the domain is more disordered compared to the wild-type domain, where *p* < 0.0001. Error bars indicate the minimum/maximum of the values obtained from the analysis of two different sequences.

We used IUPred3 to predict the disorder propensities of the regions of the PUFD domain in the wild-type and chimera proteins (Figure 4A). We predicted a slight increase in the disorder propensity, but the PUFD domain was predicted to retain its structurally ordered nature in the chimera protein. This is in agreement with the results of the secondary structure prediction.

A paired-sample *t*-test was performed to compare the IUPred3 scores (0–1) (Figure 4B) between the chimera and wild-type BCOR PUFD domains. There was a significant difference in the IUPred3 scores between the chimera (M = 0.1686, SD = 0.08402), and wild-type domains (M = 0.1162, SD = 0.06796); t(114) = 10.81, *p* < 0.0001.

**Figure 5:**
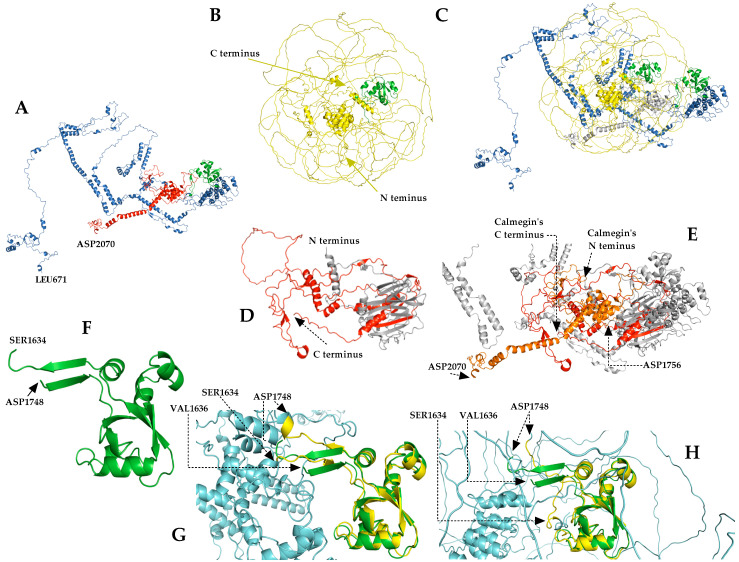
Structural comparison of the AlphaFold (AF) wild-type BCOR and calmegin protein with Robetta chimera protein, and a comparison of the wild-type experiment-determined PUFD domain with the AF- and Robetta-generated PUFD domains. (**A**): The Robetta-generated chimera protein (confidence: 0.28, range 671–2070, according to chimera numbering). The blue color indicates the amino acid sequence that corresponds to BCOR (between 671 and 1755 residues; the BCOR and chimera numbering is the same in this region) and the red color indicates the sequence corresponding to the calmegin protein (range 1756–2070, according to chimera numbering); the green color indicates the PUFD domain of BCOR (range 1634–1748, the BCOR and chimera numbering is the same in this region). (**B**): Proposed structure of the full-length BCOR protein downloaded from the AF database. The yellow color indicates the 1–1755 region of the chimera protein (being equivalent to the full-length BCOR protein), and the PUFD domain of BCOR is highlighted in green. (**C**): Aligned structures of the chimera and BCOR-aligned structure. (**D**): Structure of calmegin (total length 1–610), downloaded from AF database. The amino acid sequence corresponds to the chimera protein (from 1756 to 2070 residues). (**E**): Aligned structure of the chimera and calmegin proteins. Orange indicates the sequence of the chimera protein corresponding to calmegin. (**F**): Crystal structure of the PUFD domain of BCOR (from 1636 to 1748 residues; PDB ID: 4HPL). (**G**): Predicted structure of the PUFD domain in the chimera protein (Robetta, shown in yellow), aligned to the experimentally determined PUFD domain (PDB ID: 4HPL) (green). (**H**): Alignment of the BCOR’s PUFD domain structures, predicted by AF (yellow) and determined experimentally (PDB ID: 4HPL) (green). The light blue color indicates the non-PUFD domain residues of the chimeric protein (**G**) and AF-predicted BCOR protein (**H**).

To predict the putative 3D structure of the chimera protein, we used the Rosetta program to build a 1400 residue-long region of the complete chimera protein (671–2070 amino acids). Figure 5 shows the Robetta-constructed chimera protein (671–2070 AA; confidence: 0.28), the wild-type BCOR (1–1755 AA), and the calmegin (1–610 AA) protein, downloaded from the AF database, along with the experimentally determined PUFD domain.

**Figure 6:**
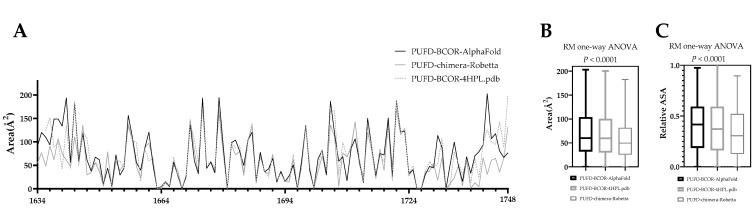
Determination of the accessible surface area (ASA) for the PUFD domain. The values were calculated, based on the predicted structures of the chimera (PUFD-chimera-Robetta) and the PUFD domain of BCOR (PUFD-BCOR-AlphaFold and PUFD-BCOR-4HPL.pdb). (**A**): The area (Å^2^)-based ASA, wherein the axis shows the residues of the PUFD domain (1634–1748 residues). (**B**): The RM one-way ANOVA significant difference calculation, based on the ASA area (Å^2^). (**C**): The RM one-way ANOVA significant difference, based on a relative ASA (0–1). A significant decrease in the PUFD-chimera-Robetta area ASA (**B**) and the relative ASA (**C**) was detected, compared to the two wild-type PUFD domains (*p* < 0.0001). The graph in part (**A**) shows the ASA values starting from the original amino acid 1634th, while the 1634th and 1635th residues were omitted from the RM one-way ANOVA statistical calculations (**B**,**C**) because these residues are missing from the PUFD-BCOR-4HPL.pdb structure (PDB ID: 4HPL).

The comparison of the ASA values of PUFD domains in wild-type (PUFD-BCOR-AlphaFold and PUFD-BCOR-4HPL.pdb) and chimera proteins (PUFD-chimera-Robetta) was carried out (Figure 6). An RM one-way ANOVA was performed to compare the relative ASA (0–1) and ASA (Å2) between PUFD-chimera-Robetta, PUFD-BCOR-AlphaFold, and PUFD-BCOR-4HPL.pdb, at between 1636 and 1748 residues. The statistical analysis was used to compare the values for 1636–1748 residues rather than for the entire PUFD domain (1634–1748 residues) because the 4HPL.pdb coordinate file does not contain the first two residues of the domain. There was a statistically significant difference in the mean score of relative ASA (0–1) (F (1.836, 205.7) = 14.28), *p* < 0.0001). Tukey’s multiple comparison tests revealed that the mean score was significantly different between the PUFD-BCOR-AlphaFold vs. PUFD-chimera-Robetta (*p* < 0.0001, 95% C.I. = [0.03140 to 0.1073]) and PUFD-BCOR-4HPL.pdb vs. PUFD-chimera-Robetta (*p* < 0.0001, 95% C.I. = [0.03103 to 0.08995]). There was no statistically significant difference in mean scores between the PUFD-BCOR-AlphaFold and the PUFD-BCOR-4HPL.pdb (0.7953).

There was also a significant difference in ASA (Å^2^) between at least two groups (F (1.859, 208.2) = 14.18), *p* < 0.0001). Tukey’s multiple comparison test found that the mean score was significantly different between the PUFD-BCOR-AlphaFold vs. the PUFD-chimera-Robetta (*p* < 0.0001, 95% C.I. = [5.832 to 19.95]), and PUFD-BCOR-4HPL.pdb vs. chimera PUFD domain (*p* < 0.0001, 95% C.I. = [5.928 to 17.06]). There was no statistically significant difference in mean scores between the PUFD-BCOR-AlphaFold and the PUFD-BCOR-4HPL.pdb (*p* = 0.8547) (Figure 6C).

Statistical analyses showed that both ASA Area 57.73 Å^2^ and relative ASA 0.3368 means were reduced in the PUFD-chimera-Robetta compared to PUFD-BCOR-AlphaFold 70.63 Å^2^, 0.4061 and PUFD-BCOR-4HPL.pdb, 69.23 Å^2^, 0.3972. It was also found that there was no significant difference in either ASA Area (Å^2^) or relative ASA, between the PUFD-BCOR-4HPL.pdb and the PUFD-BCOR-AlphaFold (Figure 6B,C). It is worth noting that the differences in structure between the terminal ends of the domain are more apparent in Figure 6A.

## 4. Discussion

BRS mainly affects children and young adults, with a wide age distribution [19], among which it has been reported that *BCOR-CCNB3*-arranged sarcoma occurred preferentially in children with skeletal distribution, whereas the alternative *BCOR*-rearranged sarcomas have more variable anatomic distribution [17]; our index fits into this clinical scenario. The differential diagnosis of the small blue round cell tumor is wide, including carcinoma, sarcoma, melanoma, and lymphoma, depending on the patient’s age, anatomical location, and specific genetic aberrations, indicating that a battery of immunohistochemical stains is usually necessary.

In our case, based on the clinical information, diagnoses of metastatic endometrioid adenocarcinoma, mediastinal small cell neuroendocrine carcinoma, and Ewing sarcoma were our initial impressions. Although our case study showed the expression of neuroendocrine markers, such as CD56 and INSM1, with negativity for cytokeratin, NUT, desmin, and SS18-SSX, the retention of the Brg1 intranuclear stain can exclude the possibility of neuroendocrine carcinoma, NUT carcinoma, rhabdomyosarcoma, poorly differentiated synovial sarcoma, and the SMARCA4 deficient thoracic tumor, respectively. CD99 and NKX2.2 positivity gave rise to the thought that it could be the Ewing sarcoma. Nevertheless, the lack of *ESWR1* gene rearrangement by FISH made us wonder whether it might be a so-called “Ewing-like” tumor. The result of the second IHC panel prompted us to consider a *BCOR*-rearranged sarcoma. During the NGS examination, we identified the novel *BCOR-CLGN* fusion. The immunohistochemical profile of the case showed similarity with other BRS; indeed, it has been reported that, from the transcriptome point of view, a distinctive cluster was found at the transcriptional level in BRS, which is different from Ewing sarcoma [42], reinforcing the concept that BRSs, either as ITD or rearrangements, exhibit a common pathogenic pathway, leading to similar morphology and immunophenotype. 

BRS was originally reported to exhibit *BCOR-ITD* and fusion with *CCNB3* within the X-chromosome, due to paracentric inversion [4]. Later, several fusion gene variants were documented, including *ZC3H7B*, *MAML3* [17], *KMT2D* [19], and, recently, *RGAG1* [18]. The oncogenic mechanisms of those fusion variants are largely unclear. Interestingly enough, in our index case, the *CLGN* is situated in proximity to *MAML3*, and both are in chromosome 4q31.1. Our NGS result identified the in-frame breaking points of *BCOR* (chrX: 39.911.366) in exon 15 and *CLGN* (chr4:141.317.359) in exon 9, respectively. To validate this finding, we performed RT-PCR and Sanger sequencing, which also revealed the in-frame fusion of both *BCOR* and *CLGN* and further verified our novel finding.

It is known that the *BCOR* gene has 16 alternative exons that regulate germinal center formation in lymph nodes and apoptosis. BCOR is also known to be a member protein of polycomb repressive complex 1.1 (PRC1.1) through its PUFD domain, in exon 15, which interacts with the RAWUL domain of the PCGF protein of PRC1.1 [16], which, in turn, although binding the non-methylated CpG islands, ubiquitylates histone 2A to repress gene expression [6]. BCOR plays an important role in tissue development and maintains mesenchymal stem cell function [43], while its high expression maintains cells in their pluripotent status [12,44]. In our case, since the coding sequences of *BCOR* were largely preserved, it might contribute a significant oncogenic role by exerting its genetic silencing effect via epigenetic regulation [45]. On the other hand, *CLGN* encodes a protein called calmegin, which is a testis-specific endoplasmic reticulum chaperone protein that plays an important role in spermatogenesis, intracellular calcium homeostasis, and the synthesis of proteins and steroid hormones. It is usually expressed in the testis, prostate, and heart. It has been documented that calmegin is transcriptionally regulated by histone deacetylase and CpG methyltransferase [46]; the deregulation of calmegin methylation capability may lead to infertility, endocrine-, prostatic- and germ cell neoplasms [47,48,49,50,51]. However, a *CLGN* translocation/fusion-associated tumor, based on our best knowledge, has not been reported as yet. We assume that the BCOR-CLGN chimeric protein reported herein may exert its oncogenic potential via the antiapoptotic effect of BCOR and epigenetic dysregulation from the calmegin.

We found that the protein product of the *BCOR*-*CLGN* fusion gene is composed of full-length BCOR protein (1–1755) and the 295–610 region of calmegin; the chimera protein consists of a total of 2070 residues. Using the ProtParam tool, the physicochemical characteristics of the wild-type BCOR protein and fusion protein were calculated and compared. By a disorder prediction using the IUPred3 web server, we found a significant increase in the PUFD domain’s disorder propensity in the chimera protein, compared to the wild-type BCOR protein’s domain. Using the Robetta webserver, a 1400 AA long region of the 2070 AA long chimera protein (encompassing its 671–2070 residues) was built up; this proposed structure was used to perform alignments to compare the folding of the chimera to those of the wild-type BCOR and calmegin proteins. The crystal structure of the PUFD domain (PDB ID: 4HPL) was also compared to the PUFD-chimera-Robetta and PUFD-BCOR-AlphaFold structures to visualize the structural differences and similarities of the domain (Figure 5). One limitation of this comparison of the 3D structures is that the Robetta web server has a 1400 residue prediction limit; therefore, it was not possible to predict the structure of the full-length chimera protein. In addition, the experimentally determined PUFD domain (PDB ID: 4HPL) lacked the 1634th and 1635th residues, which were not included in the comparison. However, the predicted structure contained the entire PUFD domain of the BCOR protein (671–1755), the breakpoint, and also the truncated calmegin (1755–2070), which made it possible to estimate the structure of the chimera and the effects of protein fusion on the PUFD domain. We assume that the 1–670 region of BCOR (which is missing from the modeled chimera structure) retains the overall structural characteristics of the wild-type BCOR upon fusion with the truncated calmegin. Although we have predicted changes in its disorder propensities, the comparison of the predicted structures revealed that the PUFD domain of BCOR may retain its globular fold in the chimera (Figure 5G). This hypothesis is in agreement with the results of the secondary structure prediction, which also implied that there would be no changes to the BCOR and calmegin proteins upon fusion.

The full-length calmegin protein contains a signal sequence at its N-terminus for translocation to the endoplasmic reticulum. This signal sequence is missing from the calmegin in the chimera protein, indicating that it is not localized in this cell compartment. Rather, IHC revealed strong intranuclear positivity for BCOR, indicating that not only the wild-type BCOR but the chimera is also localized in the nucleus.

Based on our ASA area (Figure 6B) and relative ASA (Figure 6C) analyses, the PUFD domain (especially the N- and C-terminal region) has a reduced surface accessibility in the chimera protein. Due to the lack of two residues (1634 and 1635) in the crystal structure (PDB ID: 4HPL), the ASA calculations were used to examine only the 1636 to 1748 region of the PUFD domain via an RM one-way ANOVA analysis. Increased disorder (Figure 4) and decreased ASA (Figure 6) of the PUFD domain of the chimera protein may potentially affect its interaction with non-canonical polycomb repressive complex 1 (PRC1) and the maintenance of H3K27me3. Increased disorder and decreased ASA values of the BCOR’s PUFD domain in the BCOR-calmegin chimera protein (at least at the C-terminus of the domain) can be explained by the extension of the full-length BCOR protein at its C-terminus with the 295–610 region of calmegin. In addition, the presence of the truncated calmegin in the chimera protein may potentially interfere with the allosteric properties and intermolecular interactions (including the posttranslational modifications) of the wild-type BCOR. The in silico results may be proven by future in vitro experiments and may confirm the occurrence of this hypothesized reduction in its interaction with PRC1.1.

## 5. Conclusions

In this study, we report the first case of *BCOR*-rearranged sarcoma with a novel *CLGN* fusion that shared morphological and immunophenotypical similarities with other more common fusion variants, which contribute to the continued expanding molecular subtypes of BRS. Molecular ancillary tests, such as NGS and confirmatory Sanger sequencing, serve as powerful tools to discover these unusual variants. In addition, the in silico analysis of the BCOR-CLGN fusion protein is an appropriate approach to aid in better understanding the exact pathogenesis of such a rare variant, via estimation of the fusion protein’s characteristics.

## Figures and Tables

**Table 1:** The physicochemical characteristics of the wild-type and the fusion protein. GRAVY: grand average hydropathy, AI: aliphatic index, II: instability index, EC: extinction coefficient at 280 nm, +R: the number of positively charged residues, −R: the number of negatively charged residues, TpI: the theoretical isoelectric point, Mw: molecular weight, AA: amino acid.

	GRAVY	AI	II	EC	+R	−R	TpI	Mw (Da)	Sequence Length (AA)
**BCOR-CLGN chimera**	−0.706	67.92	54.94	240,930	243	295	5.51	228,230.90	2070
**BCOR**	−0.668	68.38	55.26	160,115	203	226	6.06	192,188.64	1755
**Calmegin**	−0.734	72.74	44.52	121,850	75	132	4.57	70,038.60	610

## Data Availability

The data presented in this study are available on request from the corresponding author. The data are not publicly available to protect the rights of the patient.
